# Ritualized world relations: a Confucian critique of Rosa’s limitations on resonance

**DOI:** 10.1186/s40711-023-00184-7

**Published:** 2023-03-17

**Authors:** Geir Sigurðsson, Paul J. D’Ambrosio

**Affiliations:** 1 Chinese Studies and Philosophy, Head of Programme, Chinese Studies, School of Humanities, University of Iceland, Reykjavik, Iceland; 2 Center for Intercultural Research, Fellow of the Institute of Modern Chinese Thought, East China Normal University, 500 Dongchuan Road, Shanghai, 200241 Minhang China

**Keywords:** Resonance, Ritual, World relation, Confucianism, Agency

## Abstract

Hartmut Rosa argues that our modern and post-modern societies can be understood through the notion of dynamic stabilization—institutions require growth to maintain themselves. Part of the impetus behind the acceleration that drives dynamic stabilization is the desire to make the world more available, attainable, and accessible. On both the institutional and individual levels, this is translated into making the world more within our reach, more engineerable, predictable, and controllable. Paradoxically, success in these areas is often accompanied by the world becoming increasingly silent, cold, and unresponsive. We feel alienated or that our world relation has failed. Rosa’s solution is to reestablish resonance with the world. In this paper, we argue that his notion of resonance depends on a degree of atomic agency that muffles its own efficacy. The Confucian notion of ritual offers a more dispersed notion of agency. Rather than seeing oneself, others, and the world as distinct agents or indivisible entities, a ritualized approach sees them as mutually constitutive. It is true even on the level of agency, which drastically changes our relationship with the world.

## Introduction

Hartmut Rosa’s theory of “resonance” can be seen as an attempt to alleviate the alienation caused by the “relationlessness” of human beings and the world brought about by modern and post-modern cultures. This alienation has admittedly been a double-edged sword, as Rosa remarks, because it also “made possible the spectacular successes of scientific, technological, and economic progress” (Rosa 2020: 30). We see here a clear association with Max Weber’s influential theory of “rationalization,” providing an important key to both the exponential scientific and social advances in the modern age and a more deplorable tendency in Western capitalist practice to treat everything, including human beings, as mere instrumental means to self-centered ends. The ability to “distance” oneself from one’s object, to consider it in “abstract” or “objectified” terms, certainly enables its rationalized systematization (Weber 1988a: 1–4). However, it also has the consequence of alienating human beings from each other, social institutions, and their natural surroundings. Rosa echoes Weber arguing that through capitalist practice, everyone and everything becomes “a point of aggression” (Rosa 2020: 5) in the sense of a rationalized objective to be exploited or brought under ever-increasing degrees of control.

In desiring to grasp more of the world, we seek to make it more available, attainable, and accessible (what Rosa calls the “triple A strategy”). On both the institutional and individual levels, this is translated into making the world more within our reach or engineerable, predictable, and controllable. These projects never end. They are part of the very logic of contemporary societies, which function according to what Rosa calls dynamic stabilization. That is, institutions require growth to maintain themselves. So, the more we control the world, the more we need to increase our control. Whatever becomes available, attainable, and accessible (“triple A”), this year will be outdated next year. We are constantly required to continue these processes. Paradoxically, the more we succeed in controlling and accelerating the world, the more it seems to slip away and become lost. Our relationship with the world today is increasingly marked by silence, coldness, and unresponsiveness. Resonance is Rosa’s solution.

We will begin the paper by briefly outlining Rosa’s theory of acceleration and resonance solution. The second section will look at Weber’s understanding of China and introduce the significant Chinese notion of *guanxi* (relationships) in terms of accepting contingency. Proceeding from Weber, we will look at how Confucian thinkers consider material wealth and values are considered. It sets the grounding for section three, where we will look at early Confucian understandings of ritual alongside contemporary theories of ritual, community, and relationality. Section four will argue that Confucian concentrations of contingency, *guanxi*, and ritual offer a constructive contribution to Rosa’s resonance solution, and this is found most specifically in the notion of diffused agency. The conclusion will mention other avenues for collaborative engagement between Rosa’s thought and early Chinese philosophy, including Daoist and Neo-Daoist thought.

## Rosa’s resonance solution

Rosa understands modernity and post-modernity mainly through acceleration. Increased speed may always have been a feature of human societies, but when there is a structural requirement for speed in order to *maintain* the way things are (i.e., dynamic stabilization), a society is “modern.” Rosa succinctly states: “A society can be called modern when its mode of stabilization is dynamic.” When a society “systematically requires (material) growth, (technological) acceleration and (cultural) innovation to reproduce its structure and to maintain the institutional status quo” (Rosa 2017a), then it relies on “dynamic stabilization.” The economy is the most obvious example. For an economy to remain stable, it must constantly grow. We also find this, however, in basically every area of life: our universities need to bring in more money, attract more students, and produce more research. Professors feel this in their lives—write more papers, get more grants, attract more students, and garner better ratings (teacher evaluations). Rosa refers to a hamster wheel to describe how dynamic stabilization requires constant motion just to remain in place (Rosa 2018: 40).

Rosa suggests resonance as a different way of living that resists alienation, defining it as “a mode of being in the world” which is marked by “four crucial elements.” The first step is “affection,” that is, “the sense of the experience of being truly touched or moved”; something “touches us from the outside” (Rosa 2018: 47). It could be nature, music, or a book that “speaks,” “grasps,” or “touches” us, or, as he often says, we “hear the call of X” (Rosa 2017b). Secondly, the subject responds. This step is emotional and involves self-efficacy or agency. It begins with “emotion as the experience of responsive (as opposed to purely instrumental) self-efficacy.” As we respond, we “establish a connection” “through our own inner or outer reaction” (Rosa 2018: 47). Thirdly, as a consequence of the first two steps, we are hopefully “transformed.” Rosa links this to identity:

That resonances of this sort are vital elements of any identity-formation can be read from the fact that claims such as *after reading that book,* or *after hearing that music* or *meeting that group* or *climbing that mountain, I was a different person*, are standard ingredients of almost all (auto-)biographical accounts (ibid).

Fourthly, there is an “intrinsic moment of elusiveness, i.e., non-controllability or non-disposability” (ibid) (which is also inherent in the above three steps). “We cannot actively search for resonance, and often when we try our hardest, we fail most spectacularly. Rosa gives the example of buying the most expensive tickets for one’s favorite performers, only to come away feeling totally unmoved; or to feel bored and disconnected during the holiday gatherings” (D’Ambrosio 2020: 61). He writes, “whether or not we ‘hear the call’ is beyond our will and control” (Rosa 2018: 48).

The elusive nature of resonance means there are two necessary conditions that constitute the very possibility for it to occur. They are also exactly the reasons that motivate us to control the world. Resonance actually “*requires difference* and sometimes *opposition* and *contradiction* in order to enable real encounter[s]” (ibid). Additionally, and problematically for our contemporary approaches, “resonance cannot be stored or accumulated” (ibid).

In this essay, we will present the Confucian notion of ritual and the special type of diffused or dispersed agency associated with it to develop a collaborative approach to alienation based on the intersection of classical Confucianism and Rosa. Considering both the intellectual debt of Rosa’s theory to Weber and that Weber was one of the first to compare Western development with China, we would like to take Weber as a point of departure for comparing Rosa with early Confucianism. Much of what we find in Weber can be an excellent resource for grounding a more thorough comparison of early Confucianism and Rosa on the relationship between humans and the world.^1^

## Weber’s comparative cultural studies

We would be the first to admit that Weber’s discussion of Confucianism and Daoism is based on second-hand sinological sources that were often unreliable and are, therefore, inescapably flawed in many ways. Moreover, Weber, undeniably an intellectual giant, never had the opportunity to conduct research in China, nor did he ever learn Chinese. For these reasons, his analyses must be taken with a few grains of salt. However, we believe that his insights into the confluence of ideology and external circumstances on subjective attitudes leading to certain ways of life (*Lebensführung*) are keen, often compelling, and should not be dismissed off hand.

Weber’s focus was on Western modernization, and his comparative analysis of Chinese and Indian cultures in the *Economic Ethics of the World Religions* was mainly intended to shed light on the contingency and indeterminacy of the specific Western development of the rationalization of human practices and its flip-side, the “disenchantment of the world.” While he may have overemphasized the “magical” elements in the Confucian world that he describes, he seems to be largely correct that despite its elaborate ethical structure, Confucian ways of living did not lead to a methodically rigorous bourgeois way of life. Compared with those living within the worldview of Puritan Protestantism, Confucians did not experience an ethical tension between this and another world that pushed for a systematization of life from an inward motivation such that a rationalized homogenization of all values came to the fore. Instead of a drive to rule, dominate, and transform the world, there was a stronger tendency to adapt to the world as it is, thus transforming *oneself* (Weber 1988b: 521). Weber does not deny that Confucian philosophy is a kind of rationalism. However, it is a flexible kind of rationalism that aligns with external circumstances: “The Confucian rationalism meant rational adaptation to the world. The Puritanical rationalism: rational *domination* of the world” (ibid: 534). In this way, Confucianism works from within contingencies, whereas Puritanism sees a difference between contingent and non-contingent factors or worlds.

Confucianism is a this-worldly philosophical approach. Contingencies are not to be controlled through reference to something outside of them. Everything in early Confucian thought, from conceptions of the person and agency to discussions of human interactions and our relationship with the world, is born from and remains grounded within contingencies. This is precisely where, as we will demonstrate below, Confucian thought has the potential to contribute a structural corrective to Rosa’s “resonance” solution.

Importantly, Chinese culture produced no comparable process of rationalization that was eventually to take over virtually all aspects of life. Given Weber and Rosa’s major concerns, we should emphasize that there was no form of a domineering economic system developed in China before its encounters with the West. Commercial activities did not play as large of a role in China as they did in Europe—which saw the rise of the bourgeoisie—and a systematic rationalization of the way of life did not take place in China in any way comparable to the West. Similarly, we might say that China, prior to deep western influences, was unlikely to develop dynamic stability as the logic of its institutions.

A source of China’s native resistance to the sort of rationalization and instrumentalization of impersonal relationships, which regards human beings as equally important (or unimportant), is the relationships or personal networks modeled on the family, normally referred to as *guanxi* , which simply means “relationships.” The emphasis on understanding persons and their interactions mainly through social roles and relations is undoubtedly a major part of classical Confucianism. As Weber observes (1988b: 527), accurately enough:

The Confucian ethic left people quite deliberately in the personal relationships into which they grew naturally or those given to them through social hierarchical relations. These and only these were embellished as ethics, and ultimately no other social duties were acknowledged except the humanly created reverential duties that applied to personal relationships between persons, from duke to servant, from superior to inferior official, from father and brother to son and brother, from teacher to student, from friend to friend. In the Puritanical ethic, on the other hand, such purely personal relationships—while certainly valid and ethically regulated as long as they were not ungodly—were still somewhat suspicious, because they involved creatures.

This emphasis on *guanxi* effectively worked against the alienating objectification of human relations as they were always specific depending on the roles and relations of the persons in question. Thus, it did not lead to objectification in the political and economic spheres (ibid: 528). Contingencies dominated the view of the person, ethics, and all social interactions. The entire “world relation” as Rosa puts it is thoroughly contingent and does not seek any resource external to contingencies. In this context, a person was hardly ever (if ever) abstracted from their concrete contingencies. They were nearly always understood in terms of social roles and relationships, as well as more individual predispositions and what we would refer to today as talents, character traits, and proclivities. Taking persons as wholly constituted by their contingencies meant that it was not an option to deal with them as abstract atomic individuals whose concreteness was merely, or mostly, accidental. This latter viewpoint is the prerequisite for overly rationalized and instrumentalized human and world relations—exactly the starting point for “disenchantment” and alienation, as identified by Weber, Rosa, and so many others.

Early Confucianism not only does not think of the person or interactions in abstract ways but rather celebrates their concreteness. According to texts such as the *Analects* and *Mencius,* we need to gather as much information about contingencies as possible before assessing persons or situations. References to properties, principles, or persons as abstract only impoverish our appreciation of the exact circumstances. These texts are full of stories where gathering contingencies is paramount. The complete reliance on contingencies is present even in the most extreme cases.

One of the most famous—or notorious—passages in the *Analects* is one in which Confucius insists that if either does something reprehensible, a “father covers for his son and a son covers for his father. And being true lies in this” (*Analects* 1998: 13.18). While this sort of thinking may have been conducive to corruption and nepotism,^2^ a more charitable interpretation of the passage is that it is the duty of both father and son to induce each other to be virtuous and engage in self-cultivation. Thus, they need an opportunity to remonstrate with each other in the case of misdeeds (cf. Huang 2017: 35–36). If it is left to the impersonal authorities to punish them, the punished will, in the future, “avoid punishments but will not be without a sense of shame” (*Analects* 1998: 2.3). In other words, they will not be capable of reforming themselves. Whichever interpretation we accept, both would resist the formation of a rationalized bureaucracy that “does not take the person into account, ‘sine ira et studio,’ without hate and thus without love…” (Weber 1988b: 546).

A lack of an instrumentally rationalized way of life does not entail an inability to have stimulating effects on trade or capitalist-friendly practices. However, it would differ from the historically and culturally particular kind that was to emerge in Euro-America. In any case, it is not our place to speculate on this here, outside of simply noting that to assume that China would have developed the same type of social systems with the same logic and same world relations as developed in the west is not something we, or anyone who might enjoy this paper, should entertain.

Wealth has never been considered negatively in the Confucian tradition because it is a prerequisite for living a good and worthy life. In this context, the “good life” is entirely concrete; it is the “this world” of contingencies. Ruiping Fan (2010: 233) concisely describes:

Material rewards are accepted as generally good, so there is a pragmatist affirmation and openness to various means (such as central planning, the market, or both) as the source of monetary wealth, which is in turn a source of family and individual well-being. Confucians are this worldly in pursuing a good life and human flourishing. They work for their families within a non-Puritanical acceptance of material success in this world in which material wealth is taken as, *ceteris paribus*, good and not grounds for moral suspicion. Wealth is desirable and should be pursued, as long as one does not pursue it by violating morality.

It must be stressed, though, that in Confucian literature since early times, wealth is taken as a mere means to the end of living a virtuous and worthy life, never as an end in itself. As Confucius says in the *Analects*: “Wealth and honor are what people want, but if they are the consequence of deviating from the way ("*dao* (道)"), I would have no part in them. Poverty and disgrace are what people deplore, but if they are the consequence of staying on the way, I would not avoid them” (*Analects* 1998: 4.5). In the ancient *Book of Rites*, it is also made clear that morality and the treatment of other human beings are fundamental: “virtue is the root, wealth is the branches” (*Liji* 2006–2020: Da Xue §13).^3^

The view of wealth as no more than a means to moral self-cultivation has been consistent throughout the history of Confucianism. Wealth is not even a necessary means for self-cultivation. Yan Hui, Confucius’s favorite student and a constant positive reference throughout the *Analects,* was extremely poor (6.11). Normally, however, Confucius required that his students had some basic economic security so that their studies did not negate their ability to care for their family members’ material needs (7.7).^4^

The view that money and material possessions should be taken as means alone was further developed throughout the tradition. For instance, in Neo-Confucianism influenced by Buddhism and Daoism, the importance of reducing one’s desires for wealth or material goods was accentuated. In his *Penetrating the Scripture of Change* ("Tongshu (通书)") Zhou Dunyi, a leading figure in the development of Neo-Confucianism, argues in a manner comparable to earlier Confucians that material wealth and riches are “secondary” to the “real wealth” of a moral kind of life:

The noble person takes agreement with the Way as honor, and personal peace as wealth. Therefore he is always at peace, with nothing lacking. He regards ceremonial carriages and caps as small change; he regards gold and jade as dust. The weight [of his riches] cannot be exceeded (Adler 2014: 216).

This quote reminds of the English Puritan church leader and theologian Richard Baxter’s (1615–1691) metaphorical remark that “the care for external goods should only lie on the shoulders of the ‘saint like a light cloak, which can be thrown aside at any moment.’” In reference to this remark, Max Weber adds that “fate decreed that the cloak should become a shell as hard as steel” (Weber 1988a: 203). In other words, Weber believes that rationalized capitalism has narrowed down our valuations in such a way that considerations of monetary gain have overridden all other possibilities. Baxter and Zhou Dunyi, while not being opposed to material gain as such, seem to think that it should be of little or no significance compared with moral issues and can be easily brushed aside if it finds itself in conflict with moral values. If Weber is correct, however, this may not be as easy in Western culture as Baxter assumes.

As we will see below, the complete acceptance of contingencies as having purchase on every aspect of life means Confucian thinkers view material wealth, values, and nearly everything else, in ways that validate but also go beyond Weber’s analysis. It is precisely their views on how humans relate and world relations in general that Confucianism can effectively engage in collaboration with Rosa to reimagine approaches to curb contemporary tendencies to alienation and relationlessness.

## Confucian philosophy of ritual ("*li* (礼)")

Confucianism has not always been the same Confucianism. Officially, Confucianism was the dominating ideology in China for over two millennia. As applies to most if not all politically “successful” philosophies, it became, at times, and perhaps in particular during the last centuries before the collapse of the Chinese Imperial state in 1911, conservative, reactionary, and dogmatic. Moreover, during the long course of its reign as a state ideology, it was heavily influenced by other philosophical schools of thought, such as Legalism, Daoism, and Buddhism. Thus, there is a clear gap between the early Confucian philosophy and the Confucian Imperial ideology, and the latter was often inconsistent with the former. In this discussion, therefore, we will restrict ourselves to Confucianism as a philosophy, and we will make the case that it deliberately and explicitly proposes a way of life that avoids alienation and stimulates harmony between practitioners and their environment. Such an interpretation is far from unique and would align with the Neo-Daoist understanding of Confucianism we discussed elsewhere (cf. D’Ambrosio 2016; Sigurðsson 2021).

In a culture that has been excessively rationalized, the emphasis will be placed on gaining systematic control over all aspects of life. Society is organized in such a way as to maximize the possibility of such control. According to Rosa, it is precisely this effort that produces alienation. As he says, “we aim to experience self-efficiency not by affecting and being affected, but by instrumentalizing and manipulating other people and things” (Rosa 2020: 38). Furthermore, as he continues, modernity is “structurally driven toward making the world calculable, manageable, predictable, and controllable in every respect” (Ibid). Unfortunately, such an approach to the world is precisely what “disenchants” it, that robs things of their magic and makes them “lose their resonant quality” (ibid: 44).

In early Chinese philosophy, there is a certain acknowledgment that we can only control things to a certain extent. In addition to lacking reference to power beyond contingencies, this rests upon the underlying cosmology stipulating that reality is in a continuous flux of generative change. Everything is inherently connected and distinctive. Things are born from transformation. Since there is no reference to humans having any inborn structure or meaning outside of this world, the very starting point for human–world relations is inherently interrelated. As Chen Lai outlines: “The classical Chinese perspective starts with organic holism. Everything in the cosmos is viewed as interrelated and interdependent. Each thing’s own existence and value manifests only in its relations to other things.” (Chen 2017: 1).

Time and change are understood as penetrating all things and events, leading to their incessant creation, modification, and destruction. This expression of change and time is clear in the divinatory *Classic of Changes* (*Yijing*), especially in its ‘Appended Remarks’ (‘*Yizhuan*’), which elaborates on the cosmological background of the divinatory system. While it is most certainly incorrect that Confucius himself composed the ‘*Yizhuan*,’ as it has often been claimed, there is no doubt that it expresses Confucian cosmological views (Ren 2001: 30–31).

This view of the transformational nature of reality brings about an awareness excluding the prospect of a fixed order of things—there is no referent outside of the world upon which to model it. Nevertheless (or perhaps precisely, therefore), this perspective attaches a profound value to the ability to both predict the inevitable changes and place some control on human actions and conduct. It will certainly be taken for granted that in both cases (prediction and control), things will not follow precisely according to the model. However, such an effort provides the invaluable opportunity to, respectively, prepare for a likely sequence of events and to have reasonable expectations of other people’s conduct.

As events unfold, they are constantly checked with reference to models, which are based entirely on contingencies and therefore include the expectation of the unpredictable. According to this framework, there can be full acceptance of those aspects or changes outside the pre-established model. The models themselves, and any preferred idealized version of how things should be, are always completely embedded in contingency. Therefore, the entire approach is one where space is made for encountering new changes, unforeseen elements, and the smack of uncontrollability. It differs from the cliché “expect the unexpected.” Instead, we have reserved emptiness which will be filled with that which is, and in ways which are, unknowable.

Thus, instead of aiming at the establishment of a mathematically based rationalized system, Confucians will seek to train in themselves a *sense* for their surroundings—or, as Ni Peimin puts it, a “skill” ("*gongfu* (功夫)"), widely known in reference to martial arts where it is often romanized as “Kung Fu”). While there are fathomable patterns in reality, an openness to novelty must always be kept in mind. The inevitable gap between understanding and reality can only be bridged through a creative response that takes into account both the generalities and the specificities of the situation. Such a response is a cultivated *sense*, a sophisticated resonance ("*xin* (心)" or "*gan* (感)") that is the ripening fruit of thoughtful experience and appreciation of the roots of human association.^5^ Since Confucians have tended to place more emphasis on the social world than the natural one, they have especially sought to develop a good sense for other people, a social sense that seeks attunement to the sentiments and feelings of other persons. This counteracts alienation as a lack of the ability to respond spontaneously with one’s emotions to certain experiences. An alienated person tends to be emotionally numb to experiences and not respond to them.

Cultivating such a sense of sophisticated resonance is the outcome of Confucian learning, which combines tracing one’s cultural way or ways with an assessment of the unique situation at hand in each case.^6^ This learning largely takes place through the practice of "*li* (礼)", which is often translated as “ritual” or “ceremonies.” "*Li* (礼)" originally designated sacred rituals of a religious nature, but later came to refer to an extensive range of social or communal behavior trained and structured according to established tradition or convention. "*Li*(礼)" can, therefore, refer to the simplest of “rituals,” such as social greetings and the most elaborate ones, such as weddings, coronations, and harvest blessings. While its earlier sacred connotation was retained, the sacredness gradually came to be attached to tradition itself and more immediately to the society within which it operates. In their manifestation as established performances, "*li*(礼)"-customs are handed down from one generation to another, and they receive their authority by having been practiced for a long time and are perceived as meaningful to those who partake in them. "*Li* (礼)", then, have two important functions: they make social interactions efficacious and ease the awkwardness.

The efficacy associated with "*li* (礼)" is not a foregone conclusion. In other words, "*li* (礼)" are certainly upheld because of their traditional value; however, there is an associated recognition that ineffective or otherwise “bad” "*li* (礼)" would not be passed down. Thus, as evidence of effectiveness through time and in various circumstances, the tradition itself grants the sacred value of "*li* (礼)". "*Li* (礼) "are thus not only subject to change but are expected to change. They are not viewed as “perfect” in an otherworldly sense. Indeed, they may not even be the most (rationally) efficient—the measure of efficacy here is inclusive of tradition and appreciative of aesthetics. Their relative stability contributes greatly to their performative function—and vice versa. Dependable expectations allow for smoother social interactions, less friction, and a high degree of resonance. Within this framework alienation is primarily experienced by those alien to "*li* (礼)" or those who wish to detach themselves from them. In both cases, achieving resonance can be as easy as learning and practicing "*li* (礼)".

Again, compared with Western frameworks it should be emphasized that the convincing power of "*li* (礼)" is not sourced in any other worldly reference, nor is it to be found in, for example, economic productivity. "*Li* (礼)"-customs are of this world. They are not perfect models, but rather established patterns. They do not promise solutions to every problem, but they do provide reference for creatively addressing new issues through cultivation of skill in subjects. Throughout, the second and equally important function of “easing awkwardness” is in play.

According to Weber, Rosa, and a good deal of sociologists, philosophers, and thinkers of all kinds, the rise of economic-centered culture goes hand-in-hand with a corresponding decrease in shared social expectations.^7^ One major function of social expectations is to dictate what is considered appropriate and inappropriate. Li Zehou (2016), for example, has argued that we only need notions of justice and human rights once our shared expectations have diminished (or are violated) to the extent that some persons are at a significant loss (according to whatever metric). For the purposes of this article, another major function of social expectations can be highlighted, namely the dispelling of social awkwardness. Rituals allow people to have relatively stable and predictable interactions. They can reliably know what others will do based on circumstance and the roles of those involved. This also means the subject can know what others expect from them in return. Rituals are thus involved in two interrelated dimensions: the person themselves and persons in social interactions—including those which are outside specific ritualized spaces (or particular cultural settings).

The practice of li is largely physical and thus provides us with a bodily sense of how to comport ourselves. It is no coincidence that the Chinese characters for the body, "*ti* (体)" and "*li* (礼)", are cognates, as the latter is largely and significantly expressed through the body. It may be instructive to compare li with the elementary patterns or forms ("*xing* (型)") that must be learned in the practice of most if not all martial arts (i.e., "*gongfu*/*kung fu* (功夫)"). The forms serve both to preserve certain techniques handed down from that specific martial art tradition and to instill or drill an ability in the learner to master the technique. The only way to learn them is to repeat them, even ad nauseam, in a highly conscious and attentive manner until they can be enacted without having to think about their every component or detail. They become, so to speak, second nature, constituting an inner sense that can be triggered and externalized by the body if it happens to find itself in circumstances where their activation is desirable. Thus, for instance, in a practitioner with a highly developed sensitivity, the sudden engagement with an opponent in their vicinity will trigger the appropriate sequence of forms in such a way that they serve to come to his defense. Moreover, the forms also serve to create pertinent reactions to novel circumstances. They provide the body with knowledge of, or sense for, how to confront them.

Capturing this sense of li, Ni Peimin has advanced, as mentioned above, a “*gongfu* (功夫)" approach to Confucian philosophy. He outlines this as an “orientation toward cultivation and transformation rather than only conceptualization” (2017: xvi). Comporting one’s body and heart-mind in particular ritualized ways will, through practice, transform the person. A person thus cultivated will be appropriately responsive to unique situations as well.

The "*li* (礼)"-customs have a parallel function in the social realm. As long as the customs have been practiced and internalized, a practitioner will have acquired a practicable sense for their tradition’s spectrum of meaning. "*Li* (礼)" therefore provide one with a sense of what is appropriate with regard to the cultural tradition and therefore also of the most “effective” responses to the situations encountered in daily life; in many cases without having to ponder consciously over these situations and their solutions—this can be called the effect of “*gongfu* (功夫).” Indeed, the third century BCE Confucian thinker Xunzi suggests that “great Confucians” handle affairs as if they were moving like dancing masters to the rhythm in their social surroundings: “They move along with time, bowing or rising with the times; a thousand moves, ten thousand changes, but the tradition they follow is one and the same” (Xunzi 2006–2020: 8.20). Another metaphor closer to modern culture might be the skill of jazz musicians to improvize in harmony with an accompanying band. Such skill is hardly possible unless the musicians in question have acquired, through long and disciplined training, a profound sense for the musical tradition and culture with which they interact. It creates a kind of “flow” in Csikszentmihalyi’s sense, which Rosa understands “approximately as an experience of resonance” (2020: 55).

The rise of rationalization, individualization, and concentration on bare-bones efficacy that we find coupled with attempts to make the world more triple A, as well as controllable, see not only the rise of alienation but the (perhaps corresponding) loss of ritual.

## Revisiting rituals

Modernity has generally been antithetical to the practice of rituals. The precise reason varies from thinker to thinker, but most agree that modernity itself signifies a decay of ritual practice, ritual importance, and the role of ritual in daily life. As societies become based on atomic individuals rather than communities and socially constituted persons, the relatively thick social glue of shared expectations, practices, and norms—all of which are directly related to ritual—is increasingly replaced with a comparatively thinner ethic of rights based on isolated agents. The main moral motivation goes from a constructive view of enhancing oneself and others through viewing one’s very self and actions as inherently embedded with social encumbrances to an ethics of yielding and of not trespassing or violating the rights of others. The modern conception of morality is chiefly negative. It views people as fundamentally distinct. The rights of everyone must be respected, and what individual owe one another is minimal—certainly nothing that would substantively contribute to the moral cultivation of both parties. Today “ritual” has come to symbolize stale performances done with perfunctory effort.

The anthropologist Mary Douglas laments that “Ritual has become a bad word signifying empty conformity. We are witnessing a revolt against formalism, even against form” (Douglas 1970: 19). A consequent tendency has ensued to regard ritual as a mere series of mechanized, repetitive actions. However, recent reconsiderations indicate that such a view is in many ways misleading. In phenomenology, for example, Pierre Bourdieu has argued that from the point of view of the performer, ritualized action requires intense attention to detail for the sake of modifying the ritual action in each case according to the circumstances. Ritual is therefore a social action that hinges upon how things “play themselves out,” or as Bourdieu has pointed out, even just a polite conversation, “the seemingly most mechanical and ritualized of exchanges” requires “unceasing vigilance”.

To manage this interlocking of prepared gestures and words; the attention to every sign that is indispensable, in the use of the most ritual pleasantries, in order to be carried along by the game without getting carried away by the game beyond the game […] the art of playing on the equivocations, innuendos and unspoken implications of gestural or verbal symbolism that is required, whenever the right objective distance is in question, in order to produce ambiguous conduct that can be disowned at the slightest sign of withdrawal or refusal, and to maintain uncertainty about intentions that always hesitate between recklessness and distance, eagerness and indifference (Bourdieu 1990: 80-81).

Not unlike what the early Confucians seem to have in mind, then, ritual trains a person’s sense for the situation, to make sure that things “flow” as well as possible, and thus that human communication is characterized by *resonance*. Indeed, much of the reason rituals become so important, the reason for their promoting efficacy, ease of awkwardness, and a “*gong fu* (功夫)” of living, comes from how they communicate community. The “communication” they promote is often non-spoken, even non-directly indicated. It is communication through the shared expectations of the community. As Byung-Chul Han describes (2019: 9), “Rituals are symbolic acts. They represent and pass on the values and order on which a community is based. They bring forth a *community without communication*; today, however, *communication without community* prevails.” When there is a thick sense of shared norms, people can naturally resonate with one another without, to borrow from classical Chinese philosophical terminology, acting in ways that are *for* something ("*youwei* (有为)"). Habituation, on the individual and social levels, is key.

Returning to Bourdieu, ritual instills what he calls “body hexis,” that is to say, individual habits or characteristics which can be seen as the embodiment of the habitus, the overall system of both structured and structuring dispositions within a culture. There is therefore always a certain personalization at play that simultaneously preserves and moves forward the culture in question. Habitus “is constituted in practice [i.e., through hexis] and is always oriented through practical functions” (Bourdieu 1990: 52). The body is absolutely central to this process:

The body believes in what it plays at: it weeps if it mimes grief. It does not represent what it performs, it does not memorize the past, it enacts the past, bringing it back to life. What is “learned by body” is not something that one has, like knowledge that can be brandished, but something that one is. (Ibid: 73)

Body hexis informs deportment, the way and style in which people “carry themselves” in terms of stance, gait, gesture, etc. This is fully in line with Confucius’s remark that “without studying "*li* (礼)", one will be unable to take a stance ("*li* (立)")” (*Analects* 1998: 16:13). For Bourdieu and Confucius, the body is a mnemonic device that absorbs the basics of culture in a process of learning or socializing. “Taking a stand” or “establishing oneself” refers to how one concretely lives rituals and the personal mark they bring. It is how a person becomes who they are. In a similar vein, Han argues that “rituals bring an embodied knowledge and memory, an embodied identity, a physical connectedness” (Han 2019: 20). To use Bourdieu’s vocabulary, it is through the physical experience of bodily action that the habitus, the socially constituted basis for practices, is inculcated in a way more effective than through oral teaching—this is what Han refers to as a “community without communication.” Through the performance of (formal) actions, one not only “learns” the tradition by constructing a framework of meaningful action, but also how to make such actions one’s own, how to personalize them. The process parallels the above-mentioned forms in martial arts. Though initially learnt through constant repetition, they will eventually be appropriated as personalized responses to one’s surroundings—as long as one does not give them up. Generally speaking, then, ritualistic behavior is a form of learning in much the same way as certain technical training must take place before one acquires a truly profound sense for the task at hand, and can strictly speaking let go of the technical training. A person who has successfully internalized the spirit of a certain ritualistic practice is capable of applying it spontaneously when responding to new circumstances by adapting its primary or initially “stylized” movements to these very circumstances. By providing practitioners with a sensitivity to their surroundings, then, ritual practice is conducive to resonance and resists the alienation produced by excessive rationalization and thus objectification. Indeed, this has not gone unnoticed by Rosa himself: “Rituals establish socioculturally based axes of resonance, along which vertical (with gods, the cosmos, time and eternity), horizontal (within the social community), and diagonal (referring to things) relations of resonance can be experienced” (Rosa 2016: 297).

## Ritual response to Rosa’s resonance

Contrasting integrated ritualized societies with today’s alienated atomistic ones Han argues that rituals bring forth “a *community without communication*—where the intensity of togetherness in silent recognition provides structure and meaning—to today's *communication without community*, which does away with collective feelings and leaves individuals exposed to exploitation and manipulation by neoliberal psycho-politics.”^8^

Rosa’s solution to the problems of triple A, dynamic stabilization, and excessive desires for control is to reestablish resonance. In Han’s terms, Rosa proposes a community *with* communication. We might say Rosa seeks to reestablish communication so as to develop communities. While we generally agree that some form of resonance is likely a helpful way for orienting ourselves away from alienation, we think that Rosa’s description betrays the very nature of resonance. We take seriously Rosa’s claim that resonance is “elusive” and a “*gift*” and we would add that it resists overly detailed “scientific” accounts based on clear subject–object distinctions as well (Rosa 2020: 59). Combating the problems Rosa outlines means uprooting the fundamental contrast between subjects and the world that allows for the very possibility of the “aggression” Rosa identifies.

In his highly developed theory of resonance, Rosa says that the first step is to be “touched” or “moved” by something “from the outside.” Already we have a subject–object dichotomy which can only admit some degree of interaction. The world is “outside” the subject and thereby already conceived of as “other.” To what extent subjects or subjects and the world overlap is mediated by certain limitations. The subject already exists meaningfully before being affected from “the outside” and therefore the interaction can only ever reach specific parts of the person. Further, the potential for the world to become a point of aggression is already possible here. Even when this overlap occurs there always remain elements of the individual which are not entirely part of the world. Clashes and the risk of viewing oneself as meaningfully distinct from the world are part of this very structure. It is not an anomaly to feel cut off from the world, it is inherent to this very framework.

The Confucian ritual-based discussion of the world admits no essential distinction between atomistic subjects and the world. The person is always entirely constituted by the world, including others. Individuality, agency, and other meaningful elements (e.g., morality) are cultivated within these interactions. This allows for distinctiveness without distinction. There is nothing ever meaningfully separated from the world. Of course, something might “speak,” “grasp,” or “touch” us, but it is already always part of our self/meaning-making experience. A landscape, for example, does not exist somehow “out there” but is something that the subject is always part of as well. Traditional Chinese landscape paintings well attest this point. One can still *feel* alienated, but the solution on the Confucian ritual-based model is not to seek resonance with world that is meaningfully “other.” Rather, it is to return to the world which one is already always a part of.^9^

Rosa’s second step is similarly contestable. Again, the ritual-based view does not see the subject as responding *to* something external, but rather responding *with*. On the Confucian view persons, agency, and morality are all entirely and unequivocally contingent. They are of and only of this world. Through cultivation they become meaningfully distinctive—they can “take a stand” ("*li* (立)"), but they never become disconnected. Meaning is always cultivated within particulars. The subject’s “self-efficacy” must be derived from within interactions.

Rosa’s view of agency is highly atomic and resists the full acceptance of contingencies as deterministic. It would leave, he says, subjects without their “‘own voice’ with which to respond to” contingencies (Rosa 2020: 103). In other words, the subjects would “*always give in* “ and “would be nothing but pure “voluptuaries.” The Confucian view of agency sees it as emerging from contingencies. A sense of critical reflection is cultivated from within contingencies that can look back upon them with a critical distance. In this way the subject is entirely constituted by contingencies, remains always meaningfully within them, and yet can exercise choice, agency, and reflection. On this model resonance is less something that the subject does or seeks ("*you wei* (有为)") and more of something that the subject allows to happen through not actively responding ("*wu wei* (无为)") or not responding with certain pre-established ideas ("*cheng xin* (成心)") for how things should go.

From another perspective we can say that while Rosa’s subject remains relatively “full” ("*you* (有)") the Confucian subject is relatively empty ("*wu(* 无)"). Rosa’s subject has plans, sees the world as something external to communicate with, and must act on that world to facilitate resonance. The Confucian subject understands themselves as wholly of the world, and acts *with* it.

Returning ("*fan* (反/返)") is the way one orients themselves to act *with* the world. In some sense, we can say there is no “inter” action. Instead, we have “intra-action.” Agency on this model is dispersed among others and the world itself. The subject does not only look in to decide what should be done or how to behave, they also look out. Observing the world, critically assessing situations, and thinking in ways that are attuned with others is the pinnacle of Confucian agency. Autonomy is diffused. Choices are recognized for being entirely based in contingencies and celebrated as such. Instead of trying to reject or elevate heteronomy, the Confucian model tells us to cultivate our own agency in a way that is substantively sensitive to, and acknowledges being constituted by, contingencies. Rosa’s model seeks the subject as inherently external to—at least structurally, but also at times it seems meaningfully—contingencies. Thus, separated from the world, alienation seems inevitable. Likewise, there is a limit to the degree of resonance that might be achieved. There also seem to be temporal limits, constraints based on effort, and all sorts of other factors that might prevent resonance between a subject and a world that are essentially dissimilar. The Confucian model allows for critical distance—persons, agency, morality and the like are cultivated from contingencies—without sacrificing much in the way of the possibility for complete and total resonance with the world. We should, and *can*, engage with a ritualized world where we often, or nearly always, experience some degree of resonance.

Rosa’s third point about being transformed in unknown and unpredictable ways as the result of resonance is, from the Confucian perspective, grossly understated. As wholly constituted by contingencies and always only within them, Confucians see the subject as constantly transforming in all sorts of ways, including in unknown and unpredictable ones. Transformation is not something that happens this Tuesday afternoon, or that year, but is an ongoing process. The type of transformation that Confucians are chiefly concerned with is cultivation, or moral cultivation, which asks that one use their developed agency (critical reflection) to orient themselves in certain ways. However, this model only ever speaks of “orientations” and “trajectories” because one is always, and always will be, transforming.

Again, Rosa’s framework rests on a pre-given subject who exists, either structurally or structurally and meaningfully, and in whatever degree, outside of the world. This allows for momentary, limited transformations. Rosa notes certain events, such as reading a book, hearing music, or being in nature, mark critical changes that allow the person to say: “I became a different person.” Confucians say we are always “becoming.” As “human becomings,” changes always occur. Of course, some changes are more significant, but being attuned to the smallest, most mundane everyday transformations is what allows for daily resonance. Rosa says we can “hope to be changed” but often are not. The Confucian approach appreciates that we are always transforming and provides resources for consistent resonance.

From this perspective Confucianism can critique Rosa as describing mainly “big moment resonance.” (The emphasis in Rosa’s work is on “big moments,” but he does discuss more mundane ones). A similar critique is made of ethical discourses which mainly discuss murder, genocide, and trolley cars that most of us never experience. Confucians, viewing the world, persons, agency, and morality as all entirely contingent, do not separate the way we talk, dress, our hand gestures, and all sorts of “small” relatively “insignificant” aspects of life from moral discussion. For Confucians, if we get right how we treat our parents, siblings, and neighbors, which includes everything from clothing and vocabulary to the speed we walk or when we eat, we are less likely to have to deal with questions about murder, rape, and trolley cars out of control. Rosa’s theory of resonance is similar. What he describes are singular moments or experiences where huge changes occur. A transformation that made someone who previously experienced themselves as a relatively stable person suddenly say to themselves: “I am someone different now!” From the perspective of traditional Confucian texts this is interesting and important. But what is much more interesting and much more important is the everyday resonance we experience, along with the everyday transformations—and this all comes from recognizing that one is entirely contingent; contingent upon others and the world.

Rosa thinks resonance is often hardest to achieve when we try the most. As quoted above, he writes, “Whether or not we ‘hear the call’ is beyond our will and control” (Rosa 2018: 48). This is part-and-parcel to his emphasis on “big moment resonance.” When we really expect it and expect it in deep and meaningful ways we can easily fail. Confucians can say that this is true because we enter the situation relatively “full” ("*you* (有)")—we have pre-set expectations for how things should go—and we act for ("*you wei* (有为)") these expectations. Confucians appreciate resonance in an everyday sense and therefore do not think it is inherently difficult to achieve. They would agree with Rosa that being full of predetermined projections and acting for them will likely impede any deep and meaningful resonance. But they would add that it is more important to be resonant with the world all the time, and in all ways. We should seek resonance not so much, as Rosa emphasizes, in special events, but in everyday ones.

Accordingly, two additional points made by Rosa become muffled. With an emphasis on big life transformations Rosa will find resonance elusive; it “*requires difference* and sometimes *opposition* and *contradiction* in order to enable real encounter[s]” (Rosa 2018: 48). Likewise, “resonance cannot be stored or accumulated” (ibid). Confucians disagree. Differences can be important, but only insofar as we harmonize them. An important Confucian phrase celebrates this point “harmonizing (difference) not sameness” ("*he er bu tong* (和而不同)"). However, the Confucian understanding is less antagonistic than Rosa’s. It appreciates that there are always differences, people live different roles, hold different positions, have different dispositions and characters, but again these are all viewed as inherently interconnected, making the harmonization a constant process of mutual transformation. Secondly, traditions, including rituals, are ways to preserve and carry on effective patterns of harmonization. They are not exactly “storing” or “accumulating,” but they are methods for attempting to ensure a guarantee of resonance. While it may not always happen, there is a certain degree of likelihood. In a classroom, if students act like students, in how they think, feel, and behave, and teachers who likewise are fully teachers, resonance is likely to occur—maybe not the big life changing “I became a different person after each of my four classes today,” but “I lived fully as student and resonated with the world effectively today.”

## Conclusion

Rosa’s theory of resonance centers on distinct entities “speaking their own voice.” The concentration is on “world relations” conceived of as subjects, or subjects and world, as related in a subject–object framework. As such resonance is limited. It can only reach some parts of the subject sometimes. The emphasis is on “big moment” events—those which cause the subject to say “after x I became a different person.” The type of events Rosa discusses is often individualistic in nature, and even while change is expected on both sides, there is always a fundamental distinction between entities. Paradoxically, we cannot plan or expect resonance, though Rosa still outlines ways we can orient ourselves toward being more open to these types of experiences. Precisely for this reason we often want to store or accumulate resonance, and we are promised it in numerous ways, it cannot, however, be instrumentalized. Indeed, it is the very response to an overinstrumentalization of the world, subjects, and world relations that has led to our current feelings of alienation.

The traditional Confucian view of world relations is based on a ritualized understanding of the person, interactions, and the world. “Speaking one’s own voice” is not an overarching value. Rather, in ritual we seek to speak with others and develop a strong sense of community where our *own* voices do not always need to be heard. We speak with others through traditional roles, in particular settings, and according to shared expectations. There are no entities which are not inherently connected to all others. There is no subject–object dichotomy. All these are understood as mutually constitutive. Approaching oneself, others, and the world in this way allows us to appreciate the possibility of resonance in everyday activities. Perhaps we cannot expect it or store it, but we can enter into interactive spaces where traditional guides of effectiveness help to facilitate resonance.

Theoretically, incorporating a Confucian ritual-based perspective would allow us resources to say that we actually always are in resonance with other things. “Alienation” occurs when we do not feel a sufficient degree of resonance, or are not resonating well. But if we are constituted by others and they by us, then there is no real possibility of being disconnected from them. We need to recognize our relatedness and be more effective in our “always in resonance” state.

Concretely, the Confucian perspective can help Rosa’s theory produce more practical solutions. Currently his work remains mainly at a theoretical level with no straightforward way to envisage the application of his solution. Reimagining the place, function, and value of ritual in contemporary societies offers a concrete starting point for applying resonance theory.

## Data Availability

This is a research article. Not applicable.

## Footnotes

1 Contrary to most contemporary sinological thinkers, we believe that there is still some validity to Weber’s comparative studies of Western and Chinese cultures. Critics of Weber often accuse him of arguing in line with other German intellectuals, such as Kant and Hegel, that China lacks certain preconditions for modernization, notably rationality and a sense of transcendence (Heubel 2011and Roetz 2013). While not rejecting the validity of such a reading of his comparative study, we prefer highlighting the other side of the coin in his argument: that Chinese culture did not contain the seeds for the kind of rationalization that occurred in the Euro-American world nor its associated pernicious consequences of alienation from both nature and other people—as Weber put it himself, the “mechanized petrification, dressed up with a constipated view of one ‘s own importance” (Weber 1988a: 204). The question, however, whether it was also Weber’s intention to argue that non-Western cultures lacked components necessary for scientific, technological, economic, and social progress, lies beyond the scope of this paper.

2 We should note that while Confucianism is criticized on these grounds, for the possibility of corruption and nepotism, there is no ethical system which cannot be manipulated into having “bad” effects. The libertarian treatment of—which often amounts to completely disregarding—human rights is a great example. Contemporary ethics are not doing well today. Many are disappointed with the state of our current moral, social, and political spheres. Problems associated with COVID-19 (e.g., masking and vaccination), the environment, and economic inequality are good cases in this point.

3 For more examples from the classical Confucian literature, see Sigurðsson (2014: 135–139).

4 In *Analects* 7.7 we have Confucius saying that he required a bundle of dried meat as a form of “tuition.” Many have read this as a required fee. Most recently, for example, Raymond K. Li makes this argument (2020: 181). Fu Peirong argues that this is more of a checking mechanism to ensure that one is not ignoring the material needs of their family members (2020: 153–154).

5 The classical Chinese *xin* 心 is nowadays usually translated as “heart-mind,” but Mengzi’s use of it seems to indicate a certain sense, e.g., in his discussion of the four sprouts (*si duan* 四端) in 2A.6. *Gan* 感 is perhaps a clearer expression of such a cultivated sense or resonance, e.g., in the *Classic of Changes*: 聖人感人心而天下和平, “It is by the sage ‘s resonance with people ‘s minds that the entire empire finds peace.” Zhang Zai also summarized his philosophy of cultivation as the *gan zhi dao* 感之道, or way of sense or resonance (see on this Jia 2016: 457).

6 The notion of “cultivated sense” as the essence of learning is intriguingly close to Hans-Georg Gadamer’s understanding of the essence of *Bildung* as “a general and communal sense” (*ein allgemeiner und gemeinschaftlicher Sinn*) Gadamer (1990: 30). This comparison is elaborated in Sigurðsson (2015: 84ff).

7 There are various theories about which way the arrow of cause should point or even if this is a cause of cause and effect. Regardless, most theorists who acknowledge “modernity” as a significant shift in human cultures have highlighted capitalism (in some form or another) and loss of “tradition” (in some form or another) as constitutive to this change.

8 This is a description of the content of the English translation of Han 2019 that can be found on the website of any online bookstore.

9 Han notes something somewhat similar: “We can define rituals as *symbolic techniques of making oneself at home in the world*. They transform being-in-the-world into *being-at-home*. They turn the world into a reliable place […] they make it *habitable*. They even make it *accessible,* like a house. They structure time, furnish it” (2019: 10).
